# Methotrexate treatment strategies for rheumatoid arthritis: a scoping review on doses and administration routes

**DOI:** 10.1186/s41927-024-00381-y

**Published:** 2024-03-05

**Authors:** Esteban Rubio-Romero, César Díaz-Torné, María José Moreno-Martínez, Julen De-Luz

**Affiliations:** 1 Department of Rheumatology, Hospital Universitario Virgen del Rocío, Seville, Spain; 2 Department of Rheumatology, Hospital de la Santa Creu i Sant Pau, Universitat Autonòma de Barcelona, Barcelona, Spain; 3 Department of Rheumatology, Hospital Rafael Méndez, Lorca, Spain; 4 Gebro Pharma, Barcelona, Spain

**Keywords:** Methotrexate, Rheumatoid arthritis, Anti-rheumatic agents, Drug therapy, Clinical protocols

## Abstract

**Background:**

To describe the evidence of methotrexate (MTX) initiation strategies in patients with rheumatoid arthritis (RA) and, in the case of non-responders, analyse the efficacy and safety of route and dose optimisation.

**Methods:**

We conducted a comprehensive scoping review of randomised controlled trials according to PRISMA Scoping Reviews Checklist and the framework proposed by Arksey and O’Malley. PubMed, EMBASE, and Cochrane were searched without language restriction, and hand searches of relevant articles were examined.

**Results:**

We identified 1,367 potentially eligible studies, of which 12 were selected based on the titles and abstracts and then on the full-length articles. In naïve-MTX patients, a linear dose-response relationship for starting dose was found between 5 mg/m2/week (7.5–10 mg/week) and 10 mg/m2/week (15–22 mg/week), without toxicity correlation. A higher initial dose of MTX (25 mg vs. 15 mg) was more effective, resulting in fewer dose increases due to ineffectiveness and more dose reductions due to higher remission rates. There was also a trend towards increased gastrointestinal toxicity. Comparing different routes of administration of MTX, subcutaneous MTX showed a statistically higher ACR20 response (85%) in comparison with oral MTX (77%) (*p* < 0.05). The clinical efficacy and safety of accelerated and conventional start MTX regimens were comparable between 7.5 and 15 mg with a 2,5 mg dose increase every two weeks. In RA patients who have failed the initial treatment with MTX, the stepwise increase in MTX doses is associated with a higher ACR20 response and sustained remission rate than other strategies. In MTX non-responders, optimisation to SC MTX was associated with improvements in ACR20 and ACR50 rates with similar toxicity between groups. In the early RA patients subgroup, SC MTX showed higher ACR20 response rates than oral MTX, and intensive oral methods have a much higher sustained remission rate, shorter mean time to remission, and better clinical disease activity measures than conventional treatments.

**Conclusions:**

Higher starting doses of MTX and initial subcutaneous MTX made better performance in improving the ACR20 response, although the clinical effectiveness and safety of other MTX start regimens are comparable. This scoping review provides evidence in support of optimising MTX treatment in terms of route and dose prior to concluding that MTX treatment in RA patients has failed.

**Supplementary Information:**

The online version contains supplementary material available at 10.1186/s41927-024-00381-y.

## Introduction

Rheumatoid arthritis (RA) is a chronic inflammatory autoimmune disease characterised by pain, inflammation, and potential erosion of the joints. Approximately 0.39 to 1% of the population is affected by RA, making it one of the most prevalent chronic inflammatory diseases [1]. Unfortunately, the debilitating effects of RA have a long-term impact on patient’s physical and psychological well-being, decreasing their quality of life [2].

The primary therapeutic goal in RA is to achieve a target of sustained clinical remission or low disease activity (LDA) in each patient [3, 4], and this is typically only possible with the help of disease-modifying anti-rheumatic drugs (DMARDs). DMARDs have the potential to prevent or reduce joint damage and preserve joint integrity and function, controlling synovitis and slowing or stopping the radiographic progression [5, 6]. Various DMARDs are currently available for the treatment of RA, including conventional synthetic DMARDs (csDMARDs), biological DMARDs (bDMARDs), and targeted synthetic DMARDs [3, 4].

Among the csDMARDs, methotrexate (MTX; 4-amino-10-methyl folate acid) is currently considered the “anchor drug” for the treatment of RA [7, 8]. In fact, it is widely prescribed—for up to 70% of patients with RA [9]. With a structure similar to that of folate, this agent acts as a competitive inhibitor of multiple folate-dependent enzymes, ultimately leading to the inhibition of DNA and RNA synthesis with an increase of extracellular adenosine. Multiple mechanisms may contribute to MTX’s anti-inflammatory effects, such as purine and pyrimidine synthesis inhibition, nuclear factor-κB translocation, transmethylation reactions, Janus kinase signalling, nitric oxide production, adenosine release, and long non-coding RNA expression [10]. In the immune system, MTX primarily affects T cells, although it has also been shown to have antiproliferative or anti-inflammatory effects in B cells, monocytes, and dendritic cells [11]. In patients *naïve* to DMARDs, MTX is recommended to be part of the first treatment strategy in light of its favourable risk/benefit ratio, acceptable safety profile, and low cost [4]. It may be used as monotherapy to achieve good control or clinical remission, as well as in combination therapy for patients who require multiple DMARDs or biologic drugs to control the disease [12, 13]. Most *naïve* individuals with early RA begin oral MTX at a dosage range from 7.5 to 30 mg/week, although the optimal dose in each patient will vary because of different disease severity and pharmacokinetic variability [14]. Nevertheless, although MTX is the first choice for RA treatment, the potential side effects of MTX should not be ignored. Gastrointestinal problems, hepatotoxicity, lung toxicity, haematological toxicity, and renal toxicity are the most prevalent MTX side effects [15]. 

In an effort to minimise acute and chronic toxicity associated with MTX, new therapeutic strategies utilising different dosages and routes of administration (oral, subcutaneous, and intramuscular) have been proposed in the literature. Recent guidelines on MTX administration [4, 9] have suggested that MTX should be initiated at the highest tolerable dose (at 7.5–15 mg/week [16]), with a progressive weekly increase of the drug (to about 25 mg once weekly) to the maximal recommended dosage and an early switch to the parenteral route in case of unresponsiveness or evidence of adverse effects, before switching to another drug. However, the optimal starting dose, schedule for dose escalation, and route of administration are uncertain. First, based on the reported literature concerning the use of MTX in different dose regimens, some evidence has shown that in treatments with rapid dose escalation, the next dose increment may not result in clinically significant improvement [17] but rather increased toxicity. Furthermore, there are no specific recommendations for MTX dosage in patients with early RA since few studies have assessed the efficacy and safety of this agent in particular populations [17]. Second, regarding the different routes of administration, oral MTX is widely preferred due to patients’ better usability and lower costs [18]. However, several studies have demonstrated that the subcutaneous (SC) formulation is superior to the oral formulation in terms of discomfort and better usability by both physicians and patients [19, 20], as well as in terms of MTX bioavailability, particularly at higher doses [21–23].

Hence, considerable heterogeneity persists in the prescription of dose and methotrexate regimen for therapy of RA, with a schedule for dose escalation not fully elucidated and routes of administration not well established. Although this may be due in part to the lack of well-studied methotrexate dose-response curves and the differences in endpoints clinicians are aiming for, until now, the available literature has failed to suggest specific strategies for optimising MTX therapy, and there is no current review focusing on which are the optimal dosages and routes of administration for MTX therapy in the clinical setting. Therefore, we conducted a scoping review to describe the efficacy of using different starting doses, the schedule for dose escalation, and routes of administration to optimise the treatment of RA patients with MTX.

## Methods

The PRISMA extension for Scoping Reviews (PRISMA-ScR) [24] Checklist and the framework proposed by Arksey and O’Malley [25] were used to guide this review. The following five steps have been followed in this scoping review: (i) identifying the research question, (ii) identifying relevant studies, (iii) selecting eligible studies, (iv) charting the data, and (v) collating and summarising the results.

### Identifying the research question

The main research question was: “What are the efficacy and safety of different therapeutic strategies utilising different dosages and routes of administration of MTX in RA patients?“. The research sub-question was as follows: What are the efficacy and safety of utilising different dosages and routes of administration of MTX in individuals with early RA?

### Identifying relevant studies

A primary electronic literature search was conducted in the three major biomedical databases (PubMed, EMBASE, and Cochrane) to identify relevant publications on using different therapeutic strategies with MTX in RA patients. The search strategies were adapted for each database using a combination of free-text terms and medical subject headings (MeSH and Emtree terms). Articles published between database inception to 4th April 2022 were included in the review. In addition to this search with a more global approach aimed at patients with a time from RA diagnosis of any duration, a specific search strategy was developed to identify studies explicitly addressing the efficacy and safety of utilising different dosages and routes of administration in initial treatment with MTX only for patients early RA (disease duration ≤ 2 years). Thus, a total of two searches (one for each research question) were conducted, combining the following main search terms based on inclusion and exclusion criteria: “methotrexate”, “rheumatoid arthritis”, “drug administration routes”, “methotrexate/administration and dosage”, and “randomised controlled trial”. Most of the articles were found using combinations between Boolean operators “AND/OR”, search terms, and synonyms for the keywords.

Further search for any other relevant studies was performed through a hand search of reference lists of the selected and review articles. The searches were restricted to studies in humans, but no language or time restrictions were imposed. The final selected studies from the obtained references were screened by a single reviewer. The complete search strategies are available in Additional File 1 in the supplementary materials.

### Study selection

**Fig. 1:**
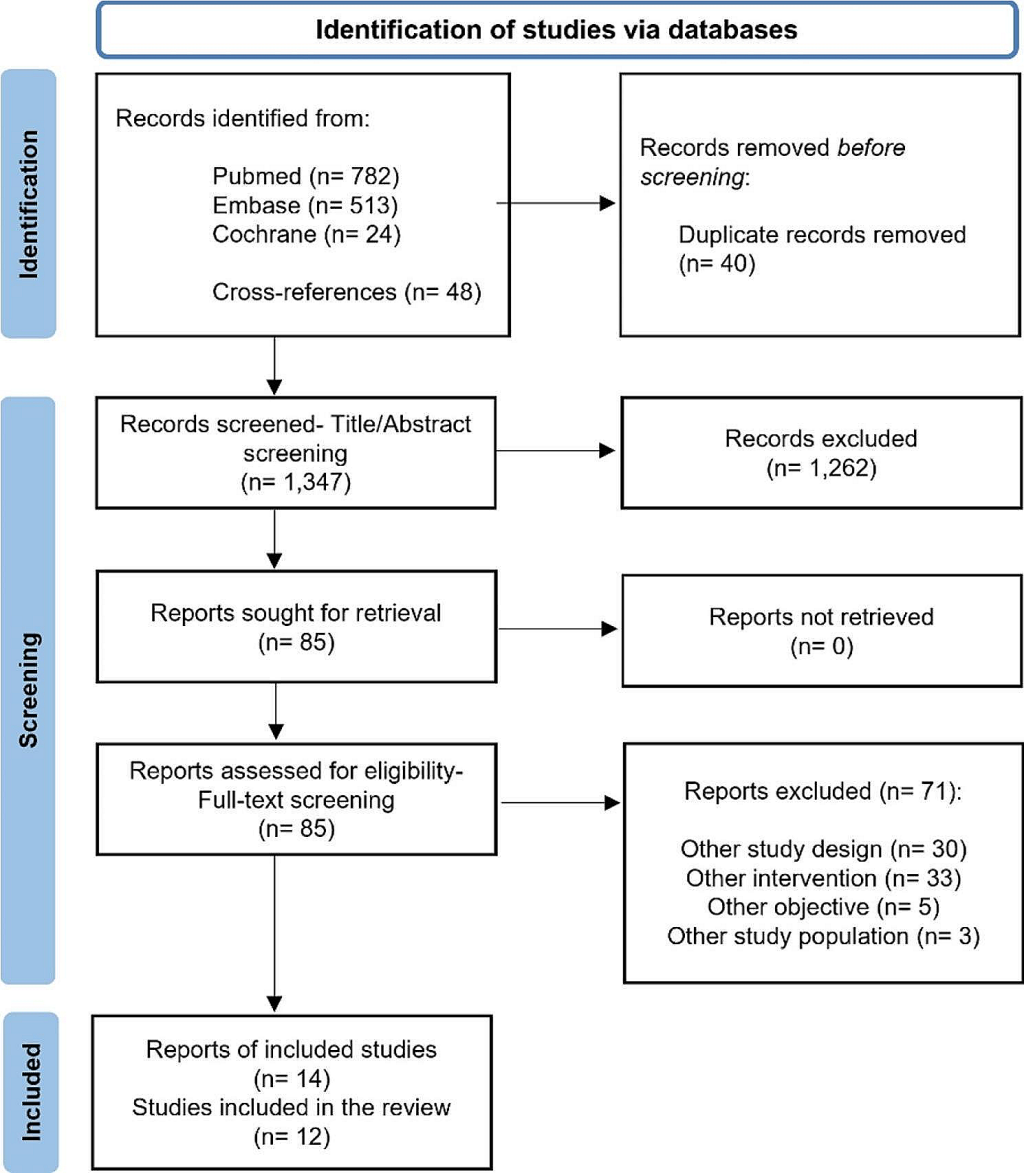
PRISMA flowchart with the main stages of the review process: primary question*. *We combined the reports based on the same research among the selected papers, resulting in 14 references covering 12 original studies

Following the manual removal of duplicates, the titles and abstracts of retrieved articles were screened for relevance. Full texts of retrieved publications were reviewed and marked for inclusion if they met the inclusion criteria. The inclusion criteria were as follows: (1) the study design was a randomised clinical trial (parallel arm and cross-over) published in any format (full paper, conference abstracts) with sufficient data available to estimate outcomes, (2) enrolled adult patients (> 18 years) with RA diagnosis according to validated criteria, irrespective of clinical stage or disease duration; and (3) compared two or more treatment strategies at treatment initiation and later on, by using different dosages or routes of administration, whether or not combined with other drugs. We consider an additional inclusion criterion on RA disease duration (≤ 2 years ) to answer the research sub-question about a specific population (early RA patients) in accordance with prior publications [26]. Papers were excluded if (1) enrolled pediatric or mixed populations, (2) focused on the use of MTX in combination with other agents or on the splitting dosing strategy (administering the total prescribed dose more frequently and in smaller increments over one week), (3) included a single arm or with any other design (narrative reviews, editorial comments, and letters). Quality appraisal was not performed in accordance with the standard approach to conducting scoping reviews [25, 27]. The PRISMA study flow diagram is illustrated in Fig. 1.

### Charting data and reporting the results

A data extraction template was developed to determine which variables to extract for each study. The following data were extracted from full-text publications selected for inclusion in the review: author(s), year of publication, study location, the title of the publication, follow-up (number of patients randomised, follow-up period, frequency of withdrawals), study population (i.e., age, gender, RA duration, baseline severity of RA, baseline functional status, MTX- or other DMARD-exposure), intervention type (i.e., treatment(s) received, dosage and dose schedule), outcome measures and critical results of the publication on the effectiveness and safety of MTX. All data were entered and verified onto a specifically designed ‘data form’ using the database program Excel. The most recent or complete report was used when multiple articles describing the same sample or study were published. We categorised the included studies based on the following three pre-identified themes: (1) initiating MTX therapy, (2) optimising MTX therapy, and (3) optimising MTX therapy in early RA patients, considering different starting doses, dose escalation strategies and routes of administration for each of them. We considered MTX therapy optimisation when the MTX dose or route of administration was modified after a failed first treatment with MTX.

## Results

### Search results

The initial electronic searches yielded 1,367 potentially eligible papers or abstracts. An additional manual search using the bibliography of select articles identified 48 more records. After excluding 1,327 duplicate references and 1,262 papers or abstracts that did not meet the eligibility criteria, 85 full-text papers were retrieved to confirm their eligibility. Of these, a further 71 articles were excluded. The main reasons for exclusion were related to inadequate study design or intervention. A total of 14 papers that met the inclusion criteria were included in this review. Among selected studies, we combined the reports based on the same research (one conference abstract and one journal article [28, 29], and two journal articles [30, 31]). Hence, finally, we included 14 references covering 12 original studies.

### Characteristics of included studies

Of the 12 trials analysing the efficacy of different MTX treatment strategies, nine sought to determine the optimal MTX dosages, while three studies [29, 30, 32] compared different MTX administration routes. Publication dates ranged from 1989 to 2021, with most studies (9/12, 75%) published since 2000. With the exception of 2 trials reported as conference abstracts [32, 33], all publications were journal articles. Except for one study with a cross-over design [31], all included studies were randomised controlled trials with a parallel longitudinal design. Most studies were conducted in Europe (*n* = 5, 41.7%), followed by Asia (*n* = 4, 33.3%) and America (*n* = 3, 25.0%). The included studies were undertaken in a single centre (including hospitals or clinics), while 4 trials were multicenter studies [31, 34–36]. Included studies were grouped into two broad categories that represent (i) the different starting dosages and the plan for dose escalation (*n* = 9) [28, 29, 33–41] and (ii) the routes of administration for MTX (*n* = 3) [30–32, 34]. A sub-analysis of findings that were unique to each category is presented below.

### Basal characteristics of patients

The 12 trials included a total of 1,566 participants. In these studies, the number of patients varied widely from 19 [38] to 384 [34]. Among included patients, 921 (58.8%) were female, with a mean follow-up of 24.3 (± 13.9) weeks, ranging from 8 to 52 weeks. All included patients fulfilled the revised American College of Rheumatology (ACR) [42] and/or the 2010 American College of Rheumatology (ACR)/EULAR classification criteria for RA [43]. The included trials recruited patients with a broad range of time from RA diagnosis (from 2.1 to 14.4 years), with a mean of 5.8 (± 4.8) years. Only two studies [28, 36] reported data on body mass index, with a mean of 24.2 (± 0.9) kg/m^2^. The mean Disease Activity Score for 28 joints (DAS28) score was reported by three trials [36, 38, 39] with scores ranging from 4.5 to 5.4 (mean 5.2 [± 0.4]), while only one study [40] provided data on the basal ACR functional class with a mean of 1.2 (± 1.3). Most subjects (921, 87.2%) were seropositive for rheumatoid factor (RF+), with percentages of RF + participants ranging from 30.4 to 85.0% among included studies. The presence of anti-cyclic citrullinated peptide (anti-CCP) was detected in 13.6% of the individuals comprising the study population. The prevalence of erosive and/or deforming arthropathy on radiographs of the hands, wrists, or feet in patients with advanced disease when this data was reported [28, 36, 39], ranged from 4.5 to 95.6%. According to data from six trials [34, 28, 36, 39–41], 353 patients (37.1%) had previous treatments with other DMARDs prior to receiving MTX. The most commonly used DMARDs were hydroxychloroquine, chloroquine, or sulfasalazine. Six studies [34, 29, 36–39] reported a total of 240 patients receiving treatment with concomitant steroids (mainly prednisone), with percentages ranging from 9.0 to 48.1% of the randomised participants. The baseline clinical characteristics of study populations are summarised in Table 1.

**Table 1:** Studies included in our review and characteristics of study populations

Author, year	Study location	Study design	Sample size	Main patient characteristics	Treatment groups	Outcomes	Study duration (weeks)
Braun, 2008 [34]	Germany	RCT, Multicenter, Double-blind, Parallel	384	Disease duration 2.3 months, Active disease, Naïve to MTX, Folic acid.	A: Oral MTX 15 mg/week with dosage adjustment after 16 weeks based on efficacy; B: SC MTX 15 mg/week with dosage adjustment after 16 weeks based on efficacy Concomitant treatment with systemic corticosteroids and NSAIDs was permitted under certain conditions, but not DMARDs or biological agents	Primary: ACR20; ESR; CRP; physician’s global assessment of disease activity; patient’s global assessment of disease activity; patient’s assessment of pain; HAQ. Secondary: ACR50; ACR70. Safety: AEs; SAEs; discontinuations due to AEs; clinical laboratory test abnormalities	24
Dhir, 2013 [28, 29]	India	RCT, Single centre, Open-label, Parallel	100	Disease duration 4.75 years, Active disease, 11% previously taken MTX, Folic acid.	A: Oral MTX 7.5 mg/week; B: Oral MTX 15 mg/week Concomitant treatement with steroids (prednisolone = < 7.5 mg/d) and other DMARDs was allowed	Primary: DAS28-3v. Secondary: Withdrawals due to any cause; withdrawals due to intolerance; patients with cytopenia or transaminitis.	12
Furst, 1989 [37]	United States	RCT, Single-center, Double-blind, Parallel	52	Active disease, Naïve to MTX, Failed DMARDs (gold, D-penicillamine).	A: Oral MTX 5 mg/m2/week; B: Oral MTX 10 mg/m2/week; C: Oral MTX 20 mg/m2/week NR concomitant treatment allowed	Diameter of the 2nd through 5th proximal interphalangeal joints of the hands; modified RAI; duration of morning stiffness; average of the last 2 of 3 grip strength; time in walk 75’ on the level; physician’s global assessment of patient’s sense of wellbeing; patient global sense of wellbeing; patient pain assessment (VAS); patient’s ability to complete 18 activities of daily living	18
Hobl, 2012 [38]	Germany	RCT, Single-center, Double-blind, Parallel	19	Active disease, Naïve to MTX, Folic acid.	A: Oral MTX 15 mg/week (standard group); B: Oral MTX 25 mg/week (accelerated group) Concomitant treatment with NSAIDs and corticosteroids was allowed.	DAS-28; swollen joint count; tender joint count; duration of morning stiffness; Patient Global Assessment; Evaluator Global Assessment; mHAQ; intensity of pain and fatigue (VAS)	16
Verstappen, 2007 [35]	Netherlands	RCT, Multicenter, Open-label	299	Naïve to DMARDs.	A: Oral MTX 7.5 mg/week, + 5 mg/month (Intensive strategy group); B: Oral MTX 7.5 mg/week + 5 mg/3 months (conventional strategy group) Concomitant treatment with NSAIDs was allowed, but not intra-articular injections or oral glucocorticoids	ESR; number of swollen joints; number of tender joints; VAS for pain; VAS general well-being; morning stiffness; HAQ17	52
Islam, 2013 [32]	India	RCT, Single centre, Parallel	92	Disease duration 4.1 years, Active disease.	A: Oral MTX; B: SC MTX NR concomitant treatment allowed	ACR20; ACR50; ACR70; AE	24
Jain, 2021 [36]	India	RCT, Multicenter, Open-label, Parallel	178	Disease duration 1.85 years, Active disease, Naïve to MTX, Folic acid.	A: Oral MTX 15 mg/week, + 5 mg/4 weeks (usual escalation group); B: Oral MTX 15 mg/week, + 5 mg/2 weeks (fast escalation group) Concomitant treatment with hydroxychloroquine, low- dose prednisolone or NSAIDs was allowed	Primary: DAS28; CRP. Secondary: DAS28- CRP; AEs and laboratory abnormalities (cytopaenias or transaminitis).	16
Lambert, 2004 [39]	United Kingdom	RCT, Single-center, Double-blind, Parallel	54	Disease duration 9.65 years, Active disease, Failed DRMARDs, and oral MTX 15–20 mg/week.	A: Oral MTX to IM 15 mg/week for 6 weeks, then: MTX IM + 5 mg/4 weeks up to 45 mg (escalation group); B: Oral MTX to IM 15 mg/week for 6 weeks, then: IM MTX 15 mg/week (control group) Concomitant treatment with 2 intraarticular steroid injections of 40 mg methylprednisolone acetate was allowed (prohibited within the final 6 weeks of the trial), but not DMARDs	Primary: DAS28 of < 3.2. Secondary: DAS28 improved by > 1.2; ACR20; % of patients achieving a good response, a moderate response, or no response; the change in the ACR core set disease activity measures; SF-12.	22
Luis, 1999 [40]	Mexico	RCT, Single-center, Single-blind, Parallel	51	Disease duration 2,0.82 years, RA in remission disease, No naïve to MTX.	A: Oral low dose MTX weekly (mean MTX dose 7.0 mg); B: Oral low dose MTX every-other-weekly (mean MTX dose 7.01 mg) Concomitant treatment with stable dosages of chloroquine or low-dose steroids was acceptable	Tender and swollen joint counts; RAI; duration of morning stiffness; HAQ; Disability Index; ACR functional class, pain (VAS); physician’s and patient’s global health assessment; AEs	24
Schiff, 2014 [30, 31]	United States	RCT, Multicenter, Open-label, Cross-over	37	Disease duration 13.3 years, Active disease, No naïve to MTX.	A: Oral MTX (10,15,20 and 25 mg/week); B: SC MTX into the abdomen; (10,15,20 and 25 mg/week); C: SC MTX into the thigh (10,15,20 and 25 mg/week) Concomitant treatment with additional medications, including DMARDs, that could interfere with PK outcome measurements was not allowed. NSAIDs were not permitted within ± 12 h of MTX administration	AUC from time 0 to the last measurable concentration (AUC0–t); or extrapolating to infinity (AUC0–inf ); maximum observed concentration (Cmax)	8
Schnabel, 1994 [41]	Germany	RCT, Single centre, Open-label, Parallel	185	Active disease, Naïve to MTX, With or without DMARDs failure.	A: Oral MTX 15 mg/week orally with dosage adjustment based on efficacy after 3 months; B: Oral MTX 25 mg/week orally with dosage adjustment based on efficacy after 3 months Concomitant treatment with analgesics, NSAIDs and low-dose methylprednisolone was allowed.	AEs; complete blood cell count; creatinine; AST; ALT and alkaline phosphatase; MTX discontinuations; withdrawals	52
Tsutsumino, 2017 [33]	Japan	RCT, Single centre, Parallel	115	Active disease, Naïve to MTX, tacrolimus, or biologics.	A: Oral MTX, + 0.25 mg/kg/week up to maximum tolerable dose; B: Oral MTX (conventional treatment group) NR concomitant treatment allowed	Primary: % of patients achieving SDAI remission and Boolean remission	24 and 48

*ACR20: American College of Rheumatology for 20% improvement; ACR50: American College of Rheumatology for 50% improvement; ACR70: American College of Rheumatology for 70% improvement; AEs: adverse events; ALT: alanine transaminase; AST: aspartate aminotransferase; CRP: C-reactive protein; DAS28-3v: disease Activity Score for 28 joints using 3 variables; DMARDs: disease-modifying anti-rheumatic drug; ESR: erythrocyte sedimentation rate; HAQ: health Assessment Questionnaire IM: intramuscular; MTX: methotrexate; NR: not reported; NSAIDs: non-steroidal anti-inflammatory drugs; RA: rheumatoid arthritis; RCT: randomised controlled trial; SAEs: severe adverse events; SC: subcutaneous; SDAI: simplified disease activity index; VAS: visual analogue scale.*

### Initiating MTX therapy

Of the 12 included trials, 7 studies [34, 29, 33, 36–38, 41] analysed the efficacy of different starting oral MTX strategies. Among them, three [29, 37, 41] compared different initial doses in a total of 337 participants (21.5%), while three trials [33, 36, 38] compared standard versus accelerated regimens for increasing oral MTX dosages on treatment initiation in a total of 312 RA patients (19.9%). Another trial [34] compared the clinical efficacy and safety of starting SC versus oral administration of MTX at 16 weeks. The main results from included RCT evaluating initial MTX therapy are shown in Table 2.

The three trials analysing the optimal oral MTX starting dose in patients with RA compared MTX doses ranging from 7.5 mg/week to 35 mg/week. Specifically, these studies compared the following MTX dosages: 5 or 10 mg/m^2^ per week [37]; 15 or 25 mg per week [41]; and 7.5 mg or 15 mg per week [28]. The first study [37] evaluated two oral MTX dosages: 5 mg/m^2^/week (7.5–10 mg/week) and 10 mg/m^2^/week (15–22 mg/week) versus placebo in 52 patients with longstanding active RA who failed other DMARDs (either gold or D-penicillamine). An additional 6 patients, given 20 mg/m^2^ MTX (27.5–35 mg/week), contributed to the toxicity but not the efficacy analysis. After 18 weeks, a linear dose-response relationship (placebo vs. 5 mg/m^2^ vs. 10 mg/m^2^) was found for 5 of 11 clinical study variables, including patient pain and global patient scale, global physician scale, joint tenderness count and activity of daily living scale (*p* < 0.05). In other words, increasing doses of MTX resulted in an increased response, and 10 mg/m^2^ (15–22 mg/week) produced more significant improvements than 5 mg/m^2^ (7.5–10 mg/week) for these outcomes. Regarding safety, despite the apparent dose-to-toxicity relationship, the authors could not find a statistically significant correlation. A second open, prospective 12-month study compared oral MTX beginning doses of 15 mg/week and 25 mg/week in 185 individuals with established RA [41]. With a few exceptions, medication was started intravenously to maximise the early effect and switched to oral after 3–4 weeks. If the therapeutic efficacy was insufficient after three months on 15 mg/week, the MTX dose was increased to 20 mg/week and, if necessary, 25 mg/week. If, after 6 months, the patient was significantly better and on a steroid dose of no more than 6 mg methylprednisolone per day, the MTX dose was permitted to be reduced in small increments (maximum 5 mg every 3 months) to the lowest effective dose. Due to ineffectiveness, 27% of patients in the 15 mg group and 3% in the 25 mg group increased their dosage. The dose was reduced in 23% and 51% of patients in the 15 and 25 mg groups, respectively, due in part to remission (10% of patients in the 15 and 35% in the 25 mg groups, respectively; *p* = 0.0001). In terms of safety, although there was a trend towards increased gastrointestinal toxicity in the higher-dosed group, the percentage of patients who reduced their dose owing to toxicity was 9% in both groups. The 12-month MTX retention rate in the 15 mg group was 74% and 73% in the 25 mg group. According to these studies, the effectiveness is highest with a high initial dose. However, in a third open-label trial [29], 100 RA patients were randomised to receive oral MTX at a starting dose of 7.5 mg or 15 mg per week, with a dosage increase of 2.5 mg every 2 weeks to a maximum of 25 mg. After 12 weeks, the authors did not find significant differences in efficacy between the 2 starting doses of MTX in terms of mean change in DAS28 (*p* = 0.60), Health Assessment Questionnaire (HAQ) score (*p* = 0.22), tender joint counts, swollen joint counts, erythrocyte sedimentation rate, patient-rated improvement, or adverse effects (transaminitis and cytopenia).

Three trials [33, 36, 38] compared standard versus accelerated regimens for increasing oral MTX concentrations in an effort to determine alternative strategies for initiating MTX administration orally. Patients in the accelerated dosing groups received oral starting doses of 15 mg/week escalated by 5 mg every 2 weeks till a maximum dose of 25 mg once a week [36]; or escalated up to 0.25 mg/kg/week within 8 weeks after the start of MTX and increased maximum tolerable dose or 16 mg/week until week 12 [33]; or 25 mg/week maintained until week 16 [38]. In standard regimen groups, the starting dose of MTX was 15 mg/week, escalated by 5 mg every 2 weeks [38] or every 4 weeks [36], or patients were treated with either MTX, tacrolimus, salazosulfapyridine, or bucillamine by the discretion of physicians until week 12 [33]. The other three trials compared MTX strategies to the fast/conventional approach, exploring intensive schedules [35], escalating doses of intramuscular (IM) MTX [39], or using different administration regimens [39].

Comparing accelerated versus standard regimens for increasing oral MTX dosages, no significant differences at 16 weeks were found for clinical efficacy variables such as DAS28 (-1.8 vs. -2.0, *p* = 0.935 [38], and − 1.3 vs. -1.3, *p* = 0.98 [36]) and HAQ score (-0.8 vs. -0.7, *p* = 0.26) [36] and (-0.8 vs. -0.56, *p* = 0.096) [33] European League Against Rheumatism (EULAR) response (65.2% vs. 61.8%, *p* = 0.64) [36], and DAS28-CRP-based remission (14.6 vs. 13.5, *p* = 0.80) [36]. Similarly, in the longer term (24 and 48 weeks), no significant differences were found between the two groups treated with oral MTX according to simplified disease activity index (SDAI) remission (42% vs. 28%, *p* = 0.1), HAQ (0 vs. 0.13, *p* = 0.096), and EuroQol-5D (0.78 vs. 0.77, *p* = 0.12) scores [33]. Considering toxicity, although significantly more gastrointestinal AE was found in the fast escalation group over the initial 8 weeks (27%, 40%, *p* = 0.048), this difference was not maintained over 16 weeks [36]. The adverse events during follow-up were generally self-limiting, and no serious adverse events were noted. There were no significant differences between the two groups related to other adverse events (such as cytopenias, transaminitis, or drug discontinuation/dose reduction).

Lastly, the multicenter trial [34] comparing the clinical efficacy and safety of SC versus oral administration of MTX at week 16 showed that ACR20 response was statistically higher in SC MTX (85%) versus 77% of those receiving oral MTX (*P* < 0.05).

**Table 2:** Initiation of MTX therapy: main findings from included RCT

Author, year	Treatment groups	Study duration (weeks)	Relevant efficacy findings	Relevant safety findings
Comparing different initial doses				
Dhir, 2013 [28, 29]	A: Oral MTX 7.5 mg/week; B: Oral MTX 15 mg/week	12	No statistically significant differences for: Mean change in DAS28 (*p* = 0.60), HAQ score (*p* = 0.22), Tender joint counts, Swollen joint counts, Erythrocyte sedimentation rate, Patient-rated improvement	No statistically significant differences for adverse effects: Transaminitis (*p* = 0.8) and, Cytopenia (*p* = 0.9).
Furst, 1989 [37]	A: Oral MTX 5 mg/m2/week; B: Oral MTX 10 mg/m2/week; C: Oral MTX 20 mg/m2/week	18	Linear dose-response relationship (placebo vs. 5 mg/m^2^ vs. 10 mg/m^2^) for: Patient pain and global patient scale, global physician scale, joint tenderness count and the activity of daily living scale (*p* < 0.05)	No statistically significant correlation between dose-to-toxicity
Schnabel, 1994 [41]	A: Oral MTX 15 mg/week orally with dosage adjustment based on efficacy after 3 months; B: Oral MTX 25 mg/week orally with dosage adjustment based on efficacy after 3 months	52	Increase dose due to ineffectiveness: 27% (15 mg group) vs. 3% (25 mg group). Dose reduction due in part to remission: 23% (15 mg group) vs. 51% (25 mg group); *p* = 0.0001.	Dose reduction due to toxicity: 9% in both groups
Comparing standard versus accelerated regimens for increasing oral MTX dosages				
Hobl, 2012 [38]	A: Oral MTX 15 mg/week (standard group); B: Oral MTX 25 mg/week (accelerated group)	16	No statistically significant differences for: DAS28 (-1.8 vs. -2.0, *p* = 0.93)	Adverse events incidence: 60% (standard dosage group) vs. 56% (accelerated dosage group). (study was not powered to detect differences)
Jain, 2021 [36]	A: Oral MTX 15 mg/week, + 5 mg/4 weeks (usual escalation group); B: Oral MTX 15 mg/week, + 5 mg/2 weeks (fast escalation group)	16	No statistically significant differences for: DAS28 (-1.3 vs. -1.3, *p* = 0.98) HAQ score (-0.8 vs. -0.7, *p* = 0.26) EULAR response (65.2% vs. 61.8%, *p* = 0.64) DAS28-CRP-based remission (14.6 vs. 13.5, *p* = 0.80)	Significantly more gastrointestinal AE in the fast escalation group at 8 weeks (27%, 40%, *p* = 0.048), but not at 16 weeks
Tsutsumino, 2017 [33]	A: Oral MTX, + 0.25 mg/kg/week up to maximum tolerable dose; B: Oral MTX (conventional treatment group)	24 and 48	No statistically significant differences for: HAQ score (-0.8 vs. -0.56, *p* = 0.096) SDAI remission (42% vs. 28%, *p* = 0.1), HAQ (0 vs. 0.13, *p* = 0.096) EuroQol-5D (0.78 vs. 0.77, *p* = 0.12)	No significant differences in incidence of severe adverse events
Comparing different routes of administration for MTX				
Braun, 2008 [34]	A: Oral MTX 15 mg/week with dosage adjustment after 16 weeks based on efficacy; B: SC MTX 15 mg/week with dosage adjustment after 16 weeks based on efficacy	24	ACR20 response was statistically higher in SC MTX (85%) versus 77% in the oral MTX group (*P* < 0.05)	

*ACR: American College of Rheumatology; DMARDs: disease-modifying anti-rheumatic drug; IM: intramuscular; MTX: methotrexate; RA: rheumatoid arthritis; RCT: randomised controlled trial; SC: subcutaneous; HAQ: Health Assessment Questionnaire; EULAR European League Against Rheumatism; DAS28: Disease Activity Score for 28 joints using 3 variables; SDAI: Simplified disease activity index.*

### Optimising MTX therapy

MTX increase and reduction dosage strategies.

Four studies [34, 35, 39, 40] evaluated different strategies for increasing MTX dosages during maintenance treatment in RA patients. The main results from included RCT analysing the optimisation of MTX therapy are shown in Table 3.

In a multicenter open-label trial [35], 299 patients with early RA were randomly assigned to either an intensive strategy with oral monthly dosage adjustments in 5 mg increments (based on the clinical response and a computerised decision tree) or a conventional approach with evaluations every 3 months by a physician. In both groups, oral administration of MTX was initiated at 7.5 mg/week, which was increased stepwise by 5 mg/week up to 30 mg/week, according to the clinical response. Individuals with suboptimal responses were switched to SC administration or given add-on cyclosporine therapy. No statistically significant differences in structural damage progression were found between the two strategies. However, the authors found a significantly higher sustained remission rate with the intensive strategy compared to the conventional approach after 1 year (35% vs. 14%, *p* < 0.001) and 2 years (50% vs. 35%, *p* = 0.03). Additionally, the mean time to sustained remission was about 4 months shorter with the intensive strategy (10.4 months vs. 14.3 months; *p* < 0.001). Another study [40] explored a method for administering oral low-dose MTX in patients with RA in remission. In this trial, 51 RA participants were randomised to continue their weekly regimen with MTX or switch to the every-other-weekly schedule. At 24 weeks, there were no significant statistical differences between the groups regarding joint counts, the Ritchie Articular Index, the HAQ score, the duration of morning stiffness, pain by visual analogue scale (VAS), or patients’ and physicians’ global health assessments (*p* value not reported). After 6 months, the incidence of adverse events did not differ statistically between groups, with the exception of a statistically significantly lower laboratory value for aspartate aminotransferase (AST) and alanine aminotransferase (ALT) in the every-other-weekly MTX group (0.041 and 0.006, respectively). Three patients relapsed, 2 taking every-other-weekly MTX, and one taking weekly MTX.

Other authors have proposed new increase dosage strategies using administration routes other than oral. In this sense, in a double-blind trial [39], 54 RA patients unresponsive to 15 mg of SC MTX were randomised to receive either 15 mg/week IM MTX with placebo dose escalation or escalation of the IM MTX dose up to 45 mg/week. The MTX or placebo dose was escalated every 4 weeks if the DAS28 was > 2.5. In the intervention arm, the dose of MTX was increased to 20 mg, 25 mg, 35 mg, and 45 mg/week consecutively every 4 weeks, while in the placebo arm, patients were administered 15 mg MTX with the addition of carrier solution at an equal volume and in colour identical to that in the intervention arm. After 22 weeks, no differences were found between groups: 1 patient (3.7%) in each group achieved a DAS28 score of < 3.2, and five patients (18.5%) in each group showed an improvement of > 1.2 in the DAS28. In addition, one patient (3.7%) in each group achieved an ACR20 response, although none achieved a good response according to EULAR response criteria. Minor adverse reactions were more frequently reported in the dose escalation group (39 vs. 29), and one patient in each group had a severe adverse reaction.

Finally, a multicenter trial [34] with 384 MTX-*naïve* participants was conducted to compare the clinical efficacy and safety of SC versus oral administration of MTX, including dosage adjustment after 16 weeks based on efficacy. Thus, at week 16, patients who did not meet the ACR20 response were switched from 15 mg of oral MTX to 15 mg of SC MTX and from 15 mg of SC MTX to 20 mg of SC MTX. A total of 52 patients (14%) were deemed ACR20 non-responders, and their medications were consequently changed per the protocol. As a result, increasing the dosage of SC MTX from 15 mg to 20 mg was associated with an ACR20 response in an additional 23% of participants.

### MTX route of administration switch strategy

Of 12 studies included in our review, two trials [32, 34] compared SC MTX versus single-dose treatment with oral MTX; one trial [31] compared oral versus SC MTX administered via an auto-injector (either into the abdomen or the thigh); and another study [39] analysed the effects of switching from oral to parenteral MTX. With the exception of one study [31] in which the authors did not specify whether initial MTX treatment failed, the patients underwent treatment with MTX for at least 3 months before randomisation; all studies included patients who had not responded adequately to initial oral MTX treatment.

The two trials [32, 34] comparing SC and oral administration routes in a total of 476 RA patients that had failed previous oral treatment found that, at 24 weeks, the rates were significantly higher in patients treated with 15 mg of SC MTX than with oral MTX for ACR20 (78% vs. 70%, *p* < 0.05 [34]; and 93% vs. 80%, *p* = 0.02 [32]) and ACR50 (89% vs. 72%, *p* = 0.03) [32]. Although one of the two trials showed response rates of ACR70 significantly higher in SC MTX than oral MTX group (41% vs. 33%, *p* < 0.05) [34], the other trial did not find that ACR70 response was significantly higher in the SC MTX group than oral group (11% vs. 9%, *p* = 0.72) [32]. Moreover, the number of swollen joints was lower in the SC group, as was the number of tender joints [34]. Further exploratory analyses stratified by disease duration showed that switching from 15 mg orally to 15 mg SC MTX when oral MTX in standard dosages was inadequately effective improved the ACR20 response in an additional 30% of patients [34]. MTX was well tolerated in these studies, and the rate of adverse events was similar in all groups [34], although the adverse effects incidence was relatively less in SC MTX. The most common side effects of SC and oral MTX were nausea (37% vs. 63%), vomiting (11% vs. 30%), dyspepsia (29% vs. 48%), dizziness (41% vs. 52%), and alopecia (72% vs. 85%), respectively [32]. Another trial comparing oral versus SC MTX administered via an auto-injector [31] showed that the mean concentration of MTX 4 h after the dose administration was consistently higher for SC MTX than for oral MTX for all dose levels but most apparent at 15–25 mg doses. Two individuals in SC MTX groups reported significant adverse events in this trial (one case of myocardial infarction with 25 mg MTX into the abdomen and one case of sick sinus syndrome with 15 mg MTX into the thigh), although no dose- or treatment-related patterns were observed. Finally, a trial [39] examined the safety and efficacy of an alternative strategy consisting of a switch to IM administration of MTX and escalation of the dose beyond conventional doses of 20–25 mg/week up to 45 mg/week. In this study, 64 patients were enrolled and were switched from 15 to 20 mg/week of oral MTX to 15 mg/week IM MTX. After 22 weeks, there was no significant difference between IM MTX and control groups in terms of change in DAS28 (-0.7 ± 1.3 vs. -0.5 ± 1.0, *p* < 0.1) or individual components of the ACR core disease activity set, and only 1 patient (3.7%) in each group achieved the primary outcome of a DAS28 < 3.2 (95% CI for the difference between the groups − 15% to + 15%).

**Table 3:** Optimisation of MTX therapy: Main findings from included RCT

Author, year	Treatment groups	Study duration (weeks)	Relevant efficacy findings	Relevant safety findings	
Comparing different MTX doses after initial failure to MTX					
Braun, 2008 [34]	A: Oral MTX 15 mg/week with dosage adjustment after 16 weeks based on efficacy; B: SC MTX 15 mg/week with dosage adjustment after 16 weeks based on efficacy	24	Increased ACR20 response in an additional 23% of participants treated with 20 mg of SC MTX		
Verstappen, 2007 [35]	A: Oral MTX 7.5 mg/week, + 5 mg/month (Intensive strategy group); B: Oral MTX 7.5 mg/week + 5 mg/3 months (conventional strategy group)	52	Statistically significantly higher sustained remission rate for the intensive strategy group at: 1 year (35% vs. 14%, *p* < 0.001), and 2 years (50% vs. 35%, *p* = 0.03). Mean time to sustained remission about 4 months shorter in the intensive strategy group (10.4 months vs. 14.3 months; *p* < 0.001).	Adverse events incidence: 87% (conventional strategy group) vs. 94% (intensive strategy group).	
Lambert, 2004 [39]	A: Oral MTX to IM 15 mg/week for 6 weeks, then: MTX IM + 5 mg/4 weeks up to 45 mg (escalation group); B: Oral MTX to IM 15 mg/week for 6 weeks, then: IM MTX 15 mg/week (control group)	22	No statistically significant differences: DAS28 score (3.7% and 18.5% in each group achieved a DAS28 score of < 3.2, and an improvement of > 1.2, respectively) ACR20 response (3.7% in each group) None achieved a good response according to EULAR response criteria.	Minor adverse reactions more common in the dose escalation group (39 vs. 29) One patient in each group had a severe adverse reaction.	
Luis, 1999 [40]	A: Oral low dose MTX weekly (mean MTX dose 7.0 mg); B: Oral low dose MTX every-other-weekly (mean MTX dose 7.01 mg)	24	No significant statistical differences for: joint counts, Ritchie Articular Index, HAQ score, duration of morning stiffness, pain by VAS, or patients’ and physicians’ global health assessments. (*p* value not reported)	A statistically significantly lower laboratory value for AST (*p* = 0,041) and ALT (*p* = 0.006) in the every-other-weekly MTX group. No significant statistical differences for adverse events incidence at 6 months	
Comparing different routes of administration for MTX after initial failure to MTX					
Braun, 2008 [34]	A: Oral MTX 15 mg/week with dosage adjustment after 16 weeks based on efficacy; B: SC MTX 15 mg/week with dosage adjustment after 16 weeks based on efficacy	24	A significantly higher response rates in SC MTX for: ACR20 (78% vs. 70%, *p* < 0.05) ACR70 (41% vs. 33%, *p* < 0.05) Number of swollen joints ((2 versus 3; *p* = 0.04)	No significant differences in adverse events incidence	
Islam, 2013 [32]	A: Oral MTX; B: SC MTX	24	Statistically significantly higher response rates in SC MTX group for: ACR20 (93% vs. 80%, *p* = 0.02) ACR50 (89% vs. 72%, *p* = 0.03) No statistically significant differences for ACR70 response (11% vs. 9%, *p* = 0.72)	Adverse effects relatively less in subcutaneous MTX (*p* value not reported)	
Lambert, 2004 [39]	A: Oral MTX to IM 15 mg/week for 6 weeks, then: MTX IM + 5 mg/4 weeks up to 45 mg (escalation group); B: Oral MTX to IM 15 mg/week for 6 weeks, then: IM MTX 15 mg/week (control group)	22	No significant differences for: Change in DAS28 (-0.7 ± 1.3 vs. -0.5 ± 1.0, *p* < 0.1) Individual components of the ACR core disease activity set the DAS28, No differences for: DAS28 < 3.2 (3.7% in each group).	Minor adverse reactions more common in the dose escalation group No significant differences for incidence of serious adverse events (1 patient in each group)	
Schiff, 2014 [30, 31]	A: Oral MTX (10,15,20 and 25 mg/week); B: SC MTX into the abdomen; (10,15,20 and 25 mg/week); C: SC MTX into the thigh (10,15,20 and 25 mg/week)	8	Mean concentration of MTX after 4 h higher for SC MTX for all dose levels (most apparent at doses of 15–25 mg)	More adverse events incidence in SC MTX group (2 vs. 0 cases)	

*ACR: American College of Rheumatology; DMARDs: disease-modifying anti-rheumatic drug; IM: intramuscular; MTX: methotrexate; RA: rheumatoid arthritis; RCT: randomised controlled trial; SC: subcutaneous; HAQ: Health Assessment Questionnaire; EULAR European League Against Rheumatism; DAS28: Disease Activity Score for 28 joints using 3 variables; SDAI: Simplified disease activity index.*

### Optimising MTX in patients with early rheumatoid arthritis

A second specific systematic search was conducted to identify studies addressing the efficacy and safety of different MTX dosages and administration routes in individuals with early RA (disease duration ≤ 2 years). Of 825 references identified, only three trials [33–35] (already identified in the broader search) fulfilled the inclusion criteria and compared different MTX regimens, specifically in patients with early RA (Additional file 2 in the Supplementary materials). The main results from included RCT evaluating the optimisation of MTX therapy in patients with early RA are shown in Table 4.

The three studies recruited 798 individuals with early RA, representing more than half of the total included population (51.0%). Only two of three trials established selection criteria related to the duration of the disease, including limits < 1 year [35] and ≤ 2 years [33], while the other trial included mixed populations (early RA and somewhat longer disease duration). In this trial [34], the mean disease duration ranged from 2.1 to 2.5 months (most patients were a population with early RA); the remaining two trials did not provide information about this data. The mean age was 56.0 years (± 2.5), and 354 patients (51.8%) were female.

Two studies analysed the results of increasing dosage strategies. The results of one of included trials compared rapid escalation and standard MTX therapy in 115 patients with early RA [33]. One group (*n* = 57) received oral doses of MTX escalated up to 0.25 mg/kg/week within 8 weeks after MTX started and increased maximum tolerable dose or 16 mg/week until week 12. The other group (*n* = 58) was managed using a conventional strategy with either MTX, tacrolimus, salazosulfapyridine, or bucillamine at physicians’ discretion until week 12. The authors found that although the rate of disease remission by SDAI criteria was higher with the rapid strategy after 24 weeks (42% vs. 28%, *p* = 0.1), these values were not statistically different between the two groups at week 48. Concerning safety, there were no significant differences between the two groups regarding the incidence of severe adverse events. Another multicenter open-label trial [35] compared an intensive treatment with oral MTX (according to a strict protocol and a computerised decision program) to conventional therapy with MTX, as described previously in the MTX dosage strategies section. As a result, a significantly higher sustained remission rate was found with the intensive strategy compared to the conventional approach after 1 year (35% vs. 14%, *p* < 0.001) and 2 years (50% vs. 35%, *p* = 0.03). Additionally, the intensive strategy approach resulted in a statistically significantly shorter mean time to sustained remission (10.4 months vs. 14.3 months; *p* < 0.001) and lower median area under the curve (AUC) for the following clinical disease activity parameters compared with the conventional strategy: morning stiffness (17.0 vs. 23.7, *p* = 0.009); erythrocyte sedimentation rate (ESR) (17.7 vs. 21.6, *p* = 0.007); tender joint count (3.6 vs. 5.5, *p* = 0.001); swollen joint count (2.7 vs. 4.7, *p* = 0.001); VAS general well-being (19.0 vs. 31.2, *p* = 0.001), and VAS pain (12.0 vs. 19.0, *p* = 0.001). Although clinical variables (including tender joint count, swollen joint count, VAS general well-being, and VAS pain) improved statistically significantly in the first year in the intensive strategy group, clinical and functional changes from baseline were similar between the two groups at two years. Response rates of ACR50 were significantly higher in the intensive strategy group at 1 year (58% vs. 43%, *p* = 0.018), but those differences did not achieve statistical significance at 2 years (46% vs. 45%, *p* = 1.00).

In a third trial [34] comparing the efficacy and safety of SC versus oral administration of MTX, the majority of patients were a population with early RA since the median time between the diagnosis of RA according to the ACR criteria and randomisation into the study was 2.1–2.5 months. In this study, ACR20 response rates over time showed a statistically significant separation between SC and oral therapy beginning as early as week 16 (85% of those receiving SC MTX versus 77% of those receiving oral MTX; *p* < 0.05).

**Table 4:** Subgroup of early RA patients: Main findings from included RCT evaluating the optimisation of MTX therapy

Author, year	Treatment groups	Study duration (weeks)	Relevant efficacy findings	Relevant safety findings
Comparing different regimens for increasing oral MTX dosages				
Tsutsumino, 2017 [33]	A: Oral MTX, + 0.25 mg/kg/week up to maximum tolerable dose; B: Oral MTX (conventional treatment group)	24 and 48	A trend to higher SDAI criteria in the rapid strategy at 24 weeks (42% vs. 28%, *p* = 0.1), but not at 48 weeks	No significant differences in adverse events incidence
Verstappen, 2007 [35]	A: Oral MTX 7.5 mg/week, + 5 mg/month (Intensive strategy group); B: Oral MTX 7.5 mg/week + 5 mg/3 months (conventional strategy group)	52	Significant differences in favour of intensive strategy group for: Sustained remission rate after 1 year (35% vs. 14%, *p* < 0.001) and 2 years (50% vs. 35%, *p* = 0.03). Mean time to sustained remission (10.4 months vs. 14.3 months; *p* < 0.001) Median AUC for morning stiffness (17.0 vs. 23.7, *p* = 0.009); erythrocyte sedimentation rate (ESR) (17.7 vs. 21.6, *p* = 0.007); tender joint count (3.6 vs. 5.5, *p* = 0.001); swollen joint count (2.7 vs. 4.7, *p* = 0.001); VAS general well-being (19.0 vs. 31.2, *p* = 0.001), and VAS pain (12.0 vs. 19.0, *p* = 0.001). No statistical differences for clinical variables (tender joint count, swollen joint count, VAS general well-being, ACR50 and VAS pain) at two years.	
Comparing different routes of administration for MTX				
Braun, 2008 [34]	A: Oral MTX 15 mg/week with dosage adjustment after 16 weeks based on efficacy; B: SC MTX 15 mg/week with dosage adjustment after 16 weeks based on efficacy	24	Significant differences in favour of the SC MTX group for ACR20 response rates as early as week 16 (85% vs. 77%; *p* < 0.05).	No significant differences in adverse events incidence

*ACR: American College of Rheumatology; DMARDs: disease-modifying anti-rheumatic drug; IM: intramuscular; MTX: methotrexate; RA: rheumatoid arthritis; RCT: randomised controlled trial; SC: subcutaneous; HAQ: Health Assessment Questionnaire; EULAR European League Against Rheumatism; DAS28: Disease Activity Score for 28 joints using 3 variables; SDAI: Simplified disease activity index.*

## Discussion

The current recommendations for using MTX in RA patients differ on important points and do not universally address all factors linked to its management. The areas of disagreement in this context include the MTX titration (the recommendations range from MTX increments of 2.5-5 mg/week every 2–6 weeks) and the starting route of MTX administration (though most recommendations supported initiating oral MTX) [44]. This scoping review summarises the available experimental evidence from the literature on the optimal dosage and route of administration of MTX in RA. The included randomised clinical trials serve as evidence for better guiding the starting and optimisation of MTX in patients with RA in daily clinical practice, considering different doses and administration routes.

Concerning the starting dose in MTX-*naïve* patients, our reviewed data generally showed a linear dose-response relationship for several clinical study variables without a significant dose-to-toxicity correlation. In particular, when comparing starting MTX doses of 7.5 or 15 mg per week, no differences are found in terms of activity disease, laboratory values, or adverse events at 12 weeks. On the other hand, comparing start doses of 15 or 25 mg per week revealed that 25 mg per week is associated with lower rates of dose reduction due in part to remission and the need for a higher dose due to ineffectiveness at 12 months, with no differences in terms of withdrawals due to side-effects between groups. Regarding the dosage increase schedule, in our study, no significant differences were found between accelerated and standard regimens for clinical efficacy and safety outcomes at short (16 weeks) or longer-term (24 and 48 weeks). The intensive strategy has been proposed in patients with early RA to take advantage of the window of opportunity that exists at this stage [45, 46]. However, the oral MTX doses in the two studies supporting these findings were greater than 15 mg. In this sense, it should be noted oral MTX absorption is limited by the saturation of the reduced folate carrier 1, a transmembrane transporter that is ubiquitous. This mechanism may become saturated if quantities of 20–25 mg are administered orally, thereby impeding absorption [47]. This mechanism could therefore explain why no significant differences were observed between the study groups. In addition, two aspects must be taken into account when interpreting these results. First, according to various abstracts presented at recent conferences [48, 49], initiating treatment with MTX at higher concentrations is associated with more rapid responses in the short term, which could consequently reduce the accumulation of damage. Second, as hypothesised by the authors, the rapid dose escalation employed by both study groups may have nullified any potential advantage of initiating treatment with a higher dose [28]. It is essential to assess the effectiveness of increasing the oral MTX dose above 15 mg without switching to the SC route. In MTX-*naïve* patients, a potential strategy could be to increase the dose of MTX and change to the SC route when doses of 15 mg are exceeded, mainly for reasons of bioavailability, decreased oral efficacy and an increased incidence of gastrointestinal side effects above these doses. In this regard, in the multicenter trial [34] comparing the clinical efficacy and tolerability of SC versus oral administration of MTX at week 16, the ACR20 response was statistically greater with SC MTX (85%) compared to oral administration (77%; P<0.05).

Concerning the optimisation of MTX dosage after initial treatment failure, the increase in MTX doses was associated with a higher ACR20 response [34], a higher sustained remission rate for 2 years, and a mean time to sustained remission that was approximately 4 months shorter than other conventional strategies [35]. The trials comparing SC and oral administration routes after the first failure to MTX revealed that SC MTX is associated with improvements in ACR20 and ACR50 rates at 24 weeks with similar adverse event rates between groups [34]. Specifically, one of the included trials [34] demonstrated that switching from 15 mg orally to 15 mg SC resulted in an ACR20 response in an additional 30% of ACR20 non-responders. Unfortunately, none of the included studies compared these results to those obtained by increasing the oral dose of MTX without switching to the SC route. Although oral MTX is widely preferred due to patient preferences and low cost [9], the greater effectiveness of the parenteral route is consistent with pharmacokinetic findings. In fact, a cross-over study in adult patients with RA showed that at doses more than 25 mg/week, oral bioavailability is 0.64 [0.21–0.96] relative to the SC route [47]. On the other hand, in RA patients in remission with MTX, no differences have been found for other proposed strategies, such as escalating IM MTX doses or different administration regimens for maintaining RA remission (weekly vs. every-other-week) [40]. 

In the subgroup of patients with early RA, compared to the conventional approach, intensive strategy approaches (oral MTX 7.5 mg/week, + 5 mg/month) resulted in a significantly higher sustained remission rate and a shorter mean time to sustained remission after 1 and 2 years, as well as an improvement in clinical disease activity parameters in the first year (but not at 2 years).

Other authors have also reviewed the available literature on the best start dosage and route of administration of MTX in patients with RA as an evidence base for generating clinical practice recommendations [23, 50, 51]. Similar to our results, those studies have pointed out that higher start MTX doses (more than 25 mg/week orally) are associated with larger clinical effects sizes, or the SC administration is more effective than oral administration with no increased adverse effects. However, our results differ slightly from those reported by Visser et al. [50]. in that fast escalation with 5 mg/month up to 25–30 mg/week was associated with larger clinical effect sizes than slow escalation. In this sense, we did not find significant differences between the two therapeutic strategies. However, these contradictory results may be due to Visser et al. included studies published from 1950 to 2007, so patients enrolled in early studies may differ from those contained in more recent studies. Moreover, the RA patients included in the different monotherapy studies exhibited a degree of heterogeneity concerning the different duration of diseases and differences in the extent of prior MTX failure, and indirect comparisons were also included. On the other side, concerning the safety of MTX and contrary to what has previously been published, we discovered no significant difference in the risk for any adverse effects between different dose regimens or routes of administration for MTX in early RA patients. However, the data on side effects in the included trials was insufficient, and more research is needed to reach a more definitive conclusion.

Our study is, up to our knowledge, the first scoping review describing analysing different starting doses, the schedule for dose escalation, and routes of administration of MTX. In our study, we aimed to find all available experimental evidence on the optimal dosage and route of administration for MTX using a strict methodological search and selection strategy. In contrast to other previous reviews, we focused our search exclusively on randomised controlled trials since this design would potentially yield the highest level of evidence without the bias generally associated with observational studies. Although the published data with this design are scant (which is a contributing factor to the limited quantity of studies retrieved by the search), they constitute the best study design for evaluating the efficacy of interventions [52]. Furthermore, our analysis was conducted based on a comprehensive search in three major databases– PubMed, EMBASE, and Cochrane Library– and we included six additional trials not included in the previously published systematic reviews and published in the period 2013–2022 with a total of 1,566 subjects. Furthermore, a comprehensive examination of the reference lists of pertinent publications was performed in order to identify any papers that may have been overlooked during the electronic search process. Our results may help to explore further the optimal start doses and route of administration of MTX, contributing to designing future treatment strategies for patients with RA. However, our work has some limitations that should be addressed. Firstly, we included only 12 trials meeting our inclusion criteria due to the lack of other available, despite the fact that MTX is widely used to treat RA in everyday practice. Secondly, we included two trials reported as conference abstracts [32, 33]. Although the information presented in conference abstracts is highly variable in reliability, accuracy, and level of detail [53], we decided to include those trials due to the limited number of randomised control trials published on the current topic. Thirdly, our review provided very little additional information regarding the rate of adverse effects in the compared groups. Considering the limitations mentioned above, we think that future prospective randomised controlled trials may significantly improve our understanding and are required to confirm our findings before any changes to therapy with MTX in everyday clinical practice could be justified. Meanwhile, our results may help to explore further the optimal start doses and route of administration of MTX, contributing to designing future treatment strategies for patients with RA.

## Conclusion

In MTX-*naïve* patients with RA, the oral starting dose demonstrates a linear dose-response relationship for multiple clinical outcomes, but there is no correlation between dose and toxicity. MTX starting doses of 7.5 or 15 mg per week were not significantly different in terms of disease activity, laboratory values, or adverse events. However, starting with doses of 25 mg was associated with a higher dose reduction rate due to an increased remission rate, compared to 15 mg MTX, which showed that the effectiveness might be highest with a high initial dose without differences in withdrawals due to adverse effects. There were no significant differences between accelerated and standard regimens in terms of clinical efficacy and safety, either in the short or long term. Regarding the administration route, SC MTX is associated with higher ACR20 response rates than oral MTX. In RA patients who have failed the initial treatment with MTX, the stepwise increase in MTX doses is associated with a higher ACR20 response, a higher sustained remission rate, and a shorter mean time to sustained remission than other conventional strategies. In these patients, SC MTX is associated with improvements in ACR20 and ACR50 rates, whereas the incidences of adverse events are comparable between groups. In the subgroup of patients with early RA, SC MTX results in higher ACR20 response rates than oral administration, and intensive oral strategies demonstrate a significantly higher sustained remission rate, a shorter mean time to sustained remission, and an improvement in clinical disease activity parameters compared to conventional approaches. Based on our findings, optimising MTX treatment in terms of route and dose prior to concluding that MTX treatment has failed may be an evidence-based therapeutic strategy for MTX in RA patients. However, this approach should always be individually adapted, taking patient characteristics, the level of disease activity, and tolerability. Our results should help to increase uniformity in medical practice and improve the management of patients treated with MTX for RA.

### Electronic supplementary material

Below is the link to the electronic supplementary material.

Supplementary Material 1

Supplementary Material 2

## Data Availability

All data related to this work are included in the main text or the supplementary materials.

## Abbreviations

ACR: American College of Rheumatology
ALT: Alanine aminotransferase
AST: Aspartate aminotransferase
AUC: Area under the curve
DAS28: Disease Activity Score for 28 joints using 3 variables
csDMARDs: Conventional synthetic disease-modifying anti-rheumatic drug
DMARDs: Disease-modifying anti-rheumatic drug
ESR: Erythrocyte sedimentation rate
EULAR: European League Against Rheumatism
HAQ: Health Assessment Questionnaire
IM: Intramuscular
LDA: Low Disease Activity
MTX: Methotrexate
RA: Rheumatoid arthritis
RF: Rheumatoid factor
RCT: Randomized controlled trial
SDAI: Simplified disease activity index
SC: Subcutaneous
VAS: Visual analogue scale
