# Supplementary material for: Methotrexate treatment strategies for rheumatoid arthritis: a scoping review on doses and administration routes
Source: BMC Rheumatol. 2024 Mar 5;8:11. doi: 10.1186/s41927-024-00381-y (PMC10913569; doi:10.1186/s41927-024-00381-y)
Supplement: Supplementary file 1 — Supplementary Material 1 [file 41927_2024_381_MOESM1_ESM.docx]

# Supplementary material (S1): Search strategies*

## Pubmed (https://pubmed.ncbi.nlm.nih.gov/):

1 "Arthritis"[Majr:NoExp] OR "rheumatoid arthritis"[ti] OR "Arthritis, Rheumatoid"[Majr:NoExp] OR rheumatoid[tw] OR rheumatism[tw] 182,354

2 Drug Administration Routes OR Drug Administration Route OR dosage OR dosing OR dose OR doses OR Drug Dose-Response Relationship OR Maximum Tolerated Dose OR Drug Administration Schedule 3,118,555

3 "Methotrexate/administration and dosage"[Majr] 3,596

4 oral OR orally OR subcutaneous OR intravenous OR intramuscular OR enteral OR parenteral OR injection OR injected 2,618,691

5 "Methotrexate"[Majr] OR Amethopterin[ti] OR Methotrexate*[ti] 22,131

6 Methotrexate OR mtx OR Amethopterin OR Methotrexat* OR Mexate* OR abitrexate* OR amethopterin* OR a-methopterin* OR ametopterin* OR antifolan* OR emtexate* OR emthexate* OR emtrexate* OR emthexate* OR folex OR ledertrexate* OR methoblastin* OR methylaminopterin* OR metotrexat* OR novatrex* OR rheumatrex 59,851

7 #6 AND #2 AND #1 4,536

8 #3 AND #1 719

9 #4 AND #5 AND #1 1,073

10 #7 OR #8 OR #9 4,773

11 Severity of Illness Index OR Treatment Outcome OR Pain Measurement OR outcome OR outcomes OR efficacy OR acr20 OR acr50 OR acr70 OR acr-20 OR acr-50 OR acr-70 OR "disease activity score" OR "disease activity scores" OR DAS[tw] OR das28 OR das44 OR das-28 OR das-44 OR sdai OR cdai OR haq[tw] OR "health assessment questionnaire" OR "health assessment questionnaires" OR ((tender OR swollen) AND count) OR "therapy response" 3,878,483

12 toxicity OR side effects OR adverse effects OR adverse events OR safety OR drug safety OR "abdominal upset" OR (upset AND (abdominal OR stomach OR gastrointestinal)) OR nausea OR anorexia OR stomatitis OR diarrhea OR liver enzymes OR liver enzyme OR alopecia OR mucositis OR leukopenia OR thrombocytopenia OR pancytopenia OR leukopaenia OR thrombocytopaenia OR pancytopaenia OR pneumonitis OR infection OR infections OR lymphoma OR lymphomas OR cirrhosis OR fibrosis OR rash OR headache OR fatigue OR malaise 8,256,000

13 #11 OR #12 10,567,777

14 #10 AND #13 4,015

15 randomized controlled trial OR controlled clinical trial OR randomized controlled trials OR random allocation OR double-blind method OR single-blind method OR clinical trial OR clinical trials OR "clinical trial" OR ((singl* OR doubl* OR trebl* OR tripl* ) AND (mask* OR blind* )) OR placebos OR placebo* OR random* OR research design [mh:noexp] OR comparative study OR evaluation studies OR cross-over studies OR randomised controlled trial OR randomised controlled trials OR groups[tiab] OR follow up studies OR follow up study OR followup OR prospective study OR prospective studies 564,588

16 #14 AND #15 782

## Embase ([www.embase.com](http://www.embase.com)):*

#1 'methotrexate'/exp OR methotrexate OR 'mtx'/exp OR mtx OR 'amethopterin'/exp OR amethopterin OR methotrexat* OR mexate* OR abitrexate* OR amethopterin* OR 'a methopterin*' OR ametopterin* OR antifolan* OR emtexate* OR emthexate* OR emtrexate* OR enthexate* OR farmitrexate* OR 'folex'/exp OR folex OR ledertrexate* OR methoblastin* OR methohexate* OR methotrate* OR methylaminopterin* OR metotrexat* OR novatrex* OR 'rheumatrex'/exp OR rheumatrex 206901

#2 'arthritis':ti OR 'rheumatoid arthritis':ti OR 'arthritis, rheumatoid':ti OR rheumatoid:ti OR rheumatism:ti 176,795*

#3 ((((drug AND administration AND routes OR drug) AND administration AND route OR dosage OR dosing OR dose OR doses OR drug) AND 'dose response' AND relationship OR maximum) AND tolerated AND dose OR drug) AND administration AND schedule 36,323*

#4 'methotrexate' OR 'administration and dosage' 210,133*

#5 oral OR orally OR subcutaneous OR intravenous OR intramuscular OR enteral OR parenteral OR injection OR injected 4,130,296*

#6 'methotrexate':ti OR amethopterin:ti OR methotrexate*:ti 25,763*

#7 #1 AND #2 AND #3 97*

#8 #2 AND #4 29,000*

#9 #2 AND #5 AND #6 2,354*

#10 #7 OR #8 OR #9 29,000*

#11 ((severity AND of AND illness AND index OR treatment) AND outcome OR pain) AND measurement OR outcome OR outcomes OR efficacy OR acr20 OR acr50 OR acr70 OR 'acr 20' OR 'acr 50' OR 'acr 70' OR 'disease activity score' OR 'disease activity scores' OR das OR das28 OR das44 OR 'das 28' OR 'das 44' OR sdai OR cdai OR haq OR 'health assessment questionnaire' OR 'health assessment questionnaires' OR ((tender OR swollen) AND count) OR 'therapy response' 5,669,600*

#12 ((((radiological AND damage OR radiographic) AND damage OR radiological) AND response OR radiographic) AND response OR 'joint damage' OR 'radiological progression' OR 'radiographic progression' OR disease) AND progression OR larsen OR sharp OR 'sharp/van der heijde' OR erosion* 815,616*

#13 ((((((toxicity OR side) AND effects OR adverse) AND effects OR adverse) AND events OR safety OR drug) AND safety OR 'abdominal upset' OR (upset AND (abdominal OR stomach OR gastrointestinal)) OR nausea OR anorexia OR stomatitis OR diarrhea OR liver) AND enzymes OR liver) AND enzyme OR alopecia OR mucositis OR leukopenia OR thrombocytopenia OR pancytopenia OR leukopaenia OR thrombocytopaenia OR pancytopaenia OR pneumonitis OR infection OR infections OR lymphoma OR lymphomas OR cirrhosis OR fibrosis OR rash OR headache OR fatigue OR malaise 4,838,417*

#14 #11 OR #12 OR #13 9,834,039*

#15 #10 AND #14 21,735*

#16 (((((((((((((((((randomized AND controlled AND trial OR controlled) AND clinical AND trial OR randomized) AND controlled AND trials OR random) AND allocation OR 'double blind') AND method OR 'single blind') AND method OR clinical) AND trial OR clinical) AND trials OR 'clinical trial' OR ((singl* OR doubl* OR trebl* OR tripl*) AND (mask* OR blind*)) OR placebos OR placebo* OR random* OR research) AND design OR comparative) AND study OR evaluation) AND studies OR 'cross over') AND studies OR randomised) AND controlled AND trial OR randomised) AND controlled AND trials OR groups OR follow) AND up AND studies OR follow) AND up AND study OR followup OR prospective) AND study OR prospective) AND studies 176,795*

#17 #15 AND #16 964*

#18 #17 AND [embase]/lim NOT ([embase]/lim AND [medline]/lim) 513

** The databases search article titles, abstracts, and full texts by default. We use field tags (usually “.ti” for title and “.ab” for abstract) to define which fields should be searched for any terms to identify the most relevant articles for our research.*

## Cochrane library (https://www.cochranelibrary.com/):

*("Arthritis" OR "rheumatoid arthritis" OR "Arthritis, Rheumatoid" OR rheumatoid OR rheumatism) AND ("early diagnosis" OR "early diagnose" OR "early diagnoses" OR "Early Detection of Disease" OR "Disease Early Detection" OR "early onset" OR "early stage" OR "early prediction" OR "early detection" OR "early treatment" OR "newly diagnosed" OR "preclinical phase" OR "at-risk of developing" OR "within 2 years" OR "<2 years duration" OR "2 years duration" OR "within 1 year" OR "<1 year duration" OR "1 year duration" OR "within 6 months" OR "<6 months duration" OR "6 months duration") in Title Abstract Keyword AND "Methotrexate" OR Amethopterin OR Methotrexate* in Title Abstract Keyword AND oral OR orally OR subcutaneous OR intravenous OR intramuscular OR enteral OR parenteral OR injection OR injected in Title Abstract Keyword AND Severity of Illness Index OR Treatment Outcome OR Pain Measurement OR outcome OR outcomes OR efficacy OR acr20 OR acr50 OR acr70 OR acr-20 OR acr-50 OR acr-70 OR "disease activity score" OR "disease activity scores" OR DAS OR das28 OR das44 OR das-28 OR das-44 OR sdai OR cdai OR haq OR "health assessment questionnaire" OR "health assessment questionnaires" OR ((tender OR swollen) AND count) OR "therapy response" in Title Abstract Keyword (Word variations have been searched)*
