# Supplementary figures and images for: Methotrexate treatment strategies for rheumatoid arthritis: a scoping review on doses and administration routes
Source: BMC Rheumatol. 2024 Mar 5;8:11. doi: 10.1186/s41927-024-00381-y (PMC10913569; doi:10.1186/s41927-024-00381-y)

# Figures

Figure S1. PRISMA Flowchart for sub-question research


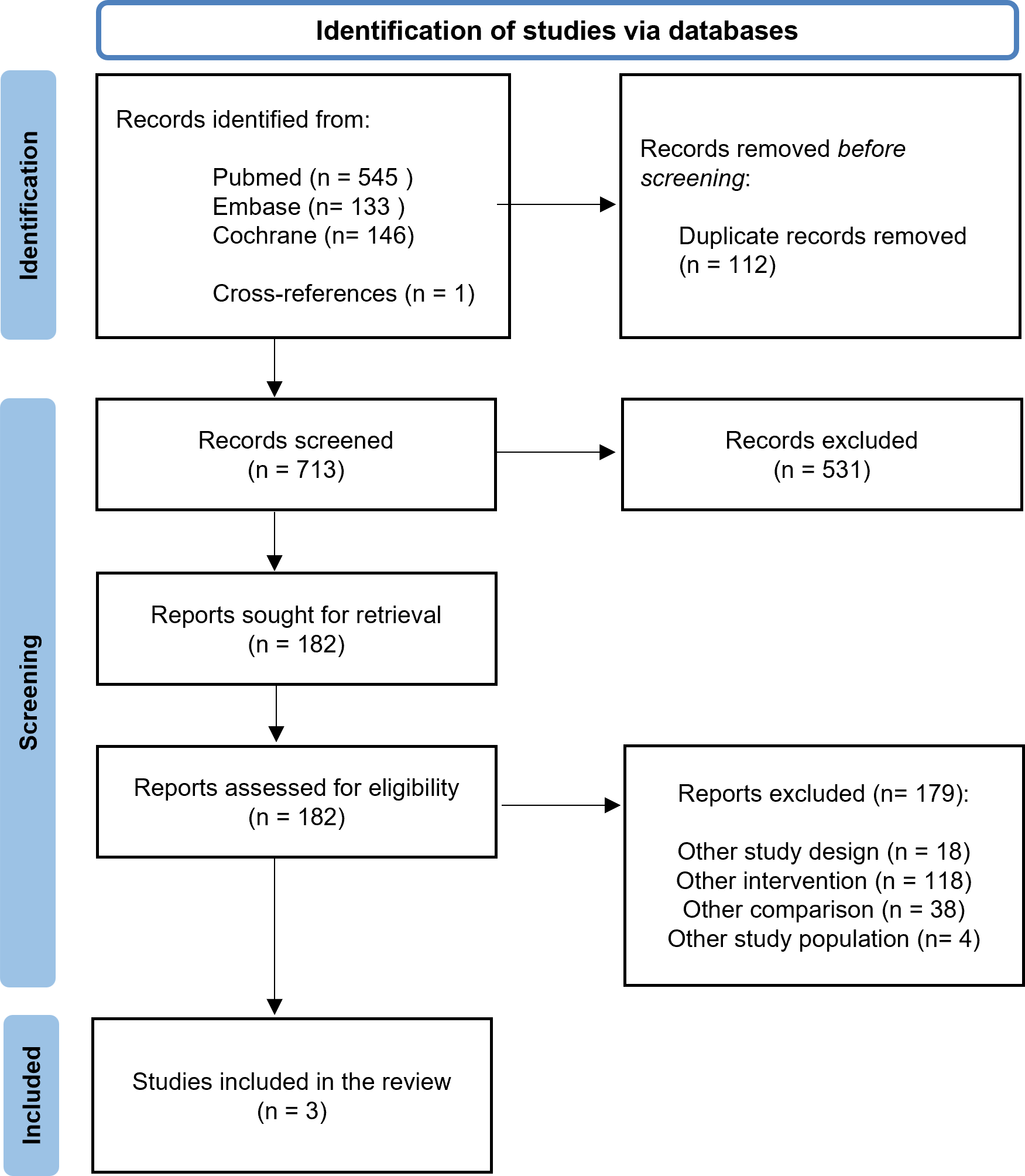

Supplement: Supplementary file 2 — Supplementary Material 2 [file 41927_2024_381_MOESM2_ESM.docx]
